# The genomic landscape of carcinomas with mucinous differentiation

**DOI:** 10.1038/s41598-021-89099-2

**Published:** 2021-05-04

**Authors:** Bastien Nguyen, Francisco Sanchez-Vega, Christopher J. Fong, Walid K. Chatila, Amir Momeni Boroujeni, Fresia Pareja, Britta Weigelt, Christos Sotiriou, Denis Larsimont, Jorge S. Reis-Filho, Christine Desmedt, Nikolaus Schultz

**Affiliations:** 1 Marie-Josée and Henry R. Kravis Center for Molecular Oncology, Memorial Sloan Kettering Cancer Center, 1275 York Ave, Box 20, New York, NY 10065 USA; 2 Human Oncology and Pathogenesis Program, Memorial Sloan Kettering Cancer Center, New York, NY 10065 USA; 3 Tri-Institutional Program in Computational Biology and Medicine, Weill Cornell Medical College, New York, NY 10065 USA; 4 Department of Pathology, Memorial Sloan Kettering Cancer Center, 1275 York Ave, New York, NY 11203 USA; 5 Breast Cancer Translational Research Laboratory J.-C. Heuson, Institut Jules Bordet, Université Libre de Bruxelles (ULB), 1000 Brussels, Belgium; 6 Department of Pathology, Institut Jules Bordet, Université Libre de Bruxelles, 1000 Brussels, Belgium; 7 Laboratory for Translational Breast Cancer Research, Department of Oncology, KU Leuven, Herestraat 49, 3000 Leuven, Belgium; 8 Department of Epidemiology & Biostatistics, Memorial Sloan Kettering Cancer Center, 1275 York Ave, New York, NY 10065 USA

**Keywords:** Cancer, Cancer genomics, Cancer

## Abstract

Mucinous carcinomas can arise in any organ with epithelial cells that produce mucus. While mucinous tumors from different organs are histologically similar, it remains to be elucidated whether they share molecular alterations. Here we analyzed a total of 902 patients across six cancer types by comparing mucinous and non-mucinous samples, integrating text mining of pathology reports, gene expression, methylation, mutational and copy-number profiling. We found that, in addition to genes involved in mucin processing and secretion, *MUC2* up-regulation is a multi-cancer biomarker of mucinous histology and is regulated by DNA methylation in colorectal, breast and stomach cancer. The majority of carcinomas with mucinous differentiation had fewer DNA copy-number alterations than non-mucinous tumors. The tumor mutational burden was lower in breast and lung with mucinous differentiation compared to their non-mucinous counterparts. We found several differences in the frequency of oncogenic gene and pathway alterations between mucinous and non-mucinous carcinomas, including a lower frequency of p53 pathway alterations in colorectal and lung cancer, and a lower frequency of PI-3-Kinase/Akt pathway alterations in breast and stomach cancer with mucinous differentiation. This study shows that carcinomas with mucinous differentiation originating from different organs share transcriptomic and genomic similarities. These results might pave the way for a more biologically relevant taxonomy for these rare cancers.

## Introduction

Mucinous carcinomas are rare histological types of cancer characterized by mucin production that can arise in any epithelial tissue that produces mucus. Mucinous tumors mostly occur in the digestive tract, including the appendix, and the breast, but also in the lung, cervix, ovaries and pancreas. Previous molecular studies were focused on individual cancer types and were often limited by sample size and the lack of multi-omics data integration^1–7^. At the transcriptomic level, *MUC2*, the most abundant secreted mucin, has been previously shown to be up-regulated in different mucinous carcinomas^1,8,9^. We and others have previously reported a lower tumor mutation burden (TMB), lower fraction of genome altered (FGA) and lower frequency of *PIK3CA* mutation in mucinous breast cancer as compared to non-mucinous breast cancer^10,11^. Other genomic studies have shown a lower FGA and a lower frequency of *TP53* mutations in mucinous colorectal carcinoma^12,13^. Despite their histological similarities, it is not known whether mucinous carcinomas from different cancer types share molecular features. Therefore, re-analysis of publicly available datasets that provides multi-omics data, represents a great opportunity to investigate the complex biology of mucinous tumors across multiple layers. Here, we investigated the molecular landscape of carcinomas with mucinous differentiation from six different cancer types by integrating text mining of pathology reports, gene expression, methylation and mutational profiling from The Cancer Genome Atlas (TCGA).

## Methods

### Data acquisition, patients and study design

All data were obtained from the TCGA Pan-Cancer Atlas (PanCanAtlas)^14,15^ dataset available at (https://gdc.cancer.gov/about-data/publications/pancanatlas). All available pathology reports from TCGA were downloaded using GDC Data Transfer Tool (https://gdc.cancer.gov/access-data/gdc-data-transfer-tool). The curated genomic alteration matrices were obtained from Sanchez-Vega et al.^15^. We used a machine learning optical character recognition method using the tesseract R package to convert the scanned pathology reports into a text file and used ontology-based text mining to identify carcinomas with mucinous features. We manually inspected every pathology report that included the following keywords; “mucin”, “mucous” and “colloid”. Whenever possible, the Hematoxylin and eosin (H&E) slides corresponding to the identified mucinous cases (143/233) were manually reviewed by a trained pathologist (D.L.). Except for CEAD, which did not have sufficient controls, each mucinous case was matched to 3 non-mucinous counterpart controls (corresponding histotype) according to gender (except for BRCA and CESC, which were all female), age at diagnosis, date of diagnosis and pathological tumor-node-metastasis (pTNM) staging. We used additional matching rules such as breast cancer subtype (ER, PR and HER2 status) for BRCA, tobacco smoking history for LUAD, organ site (colon vs. rectum) and laterality (left vs. right) for CRC. The matching was performed using the nearest neighbor matching algorithm as implemented in the MatchIt R package^16^. A total of 902 samples were included, representing 285 colorectal adenocarcinoma (CRC), 95 colorectal mucinous adenocarcinoma (CRC-muc), 108 infiltrating ductal carcinoma breast cancer (BRCA), 36 mucinous carcinomas of the breast (BRCA-muc), 108 lung adenocarcinoma (LUAD), 36 mucinous (colloid) adenocarcinoma of the lung, 99 stomach adenocarcinoma (STAD), 33 mucinous adenocarcinoma of the stomach (STAD-muc), 24 endocervical adenocarcinoma (CEAD), 18 mucinous adenocarcinoma of endocervical type (CEAD-muc), 45 pancreas-adenocarcinoma ductal type (PAAD) and 15 pancreas-colloid (mucinous non-cystic) carcinoma (PAAD-muc).

### Methylation analysis

We used data generated from Human Methylation 450 K arrays (Illumina, San Diego, CA). DNA methylation data was processed in R using the *minfi* package. Beta-values were normalized (preprocessQuantile) and the function dmpFinder (option; “shrinkVar = TRUE, type = categorical”), which use an F-test, was used to identify differentially methylated positions between carcinomas-muc and their non-mucinous counterparts within each cancer type. Differentially methylated CpGs were identified using a cut-off of FDR < 0.05. Methylation probes were mapped to genes using the illuminaHumanMethylation450kanno.ilmn12.hg19 Bioconductor package^17^.

### Mucins gene expression signatures

As previously reported^11^, the metagene signature of gel-forming (“Gel-MUC”) and membrane-bound (“Membrane-MUC”) mucins was calculated by taking the mean RPKM expression level of (*MUC2*, *MUC5B*, *MUC5AC*, *MUC6*, *MUC19*) and (*MUC1*, *MUC3A*, *MUC3B*, *MUC4*, *MUC12*, *MUC13*, *MUC14*, *MUC15*, *MUC16*, *MUC17*, *MUC20*, *MUC21*, *MUC22*) respectively, scaled to a standard deviation of one and centered around zero.

### Genomic analysis

For each cancer type, recurrent oncogenic alterations were defined as being oncogenic as per OncoKB annotation^18^ (version August 28, 2019) and present in at least 1%. Tumor mutation burden was calculated for each sample as the total number of non-synonymous mutations divided by the number of bases sequenced. Segmented copy number data were processed using CNtools package v1.4. Fraction of genome altered was calculated for each sample as the percentage of genome with log2 copy ratios > 0.2 or < − 0.2. Thresholds for copy number gain and loss were set at log2 copy ratios of > 0.2 and < − 0.2, respectively. The canonical oncogenic pathway level alterations were computed using the curated pathway templates provided in Sanchez-Vega et al.^15^.

### Statistical analysis

Differences in clinicopathological characteristics between groups were analyzed using the χ^2^ test or the Fisher exact test when appropriate. All statistical tests comparing groups were done using the non-parametric Mann–Whitney *U* test and the χ^2^ test or the Fisher exact test when appropriate for continuous and categorical variables, respectively. All correlations were calculated using the non-parametric Spearman’s rho coefficient. Overall survival (OS) and progression-free interval (PFI) was obtained for each patient from the TCGA Pan-Cancer Clinical Data Resource^19^. Survival curves were analyzed using the Kaplan–Meier method and compared by the log-rank test. The prognostic impact of mucinous features on OS and PFI was evaluated using univariable and multivariable Cox proportional hazards regression models and expressed as hazard ratio (HR) with 95% confidence interval (95CI). Multivariable analyses were adjusted for standard clinical prognostic factors (age at diagnosis (dichotomized using the median), year of diagnosis (dichotomized using the median), pathological size, node involvement and metastasis status). All interaction and multivariable tests were performed using analysis of variance to compare the models with and without the extra term. Differential expression analysis was performed with DESeq2 v.1.14.1 R/Bioconductor package^20^ on raw count data. Significantly differentially expressed genes were selected with an FDR < 0.05. To estimate the probability of observing the 102 genes (*N)* that were consistently differentially expressed between carcinomas-muc and controls across cancer type, we used a Monte-Carlo simulation. We simulated 10^5^ scenarios in which the differentially expressed genes for each cancer type were randomly permuted and intersected.

Finally, the empirical p-value was calculated by using the Monte-Carlo procedure^21^

$$p= \frac{(r+1)}{(n+1)}$$

where the *r* is the number of simulations that produced at least *N* genes and *n* is the number simulations.

Gene ontology enrichment analysis was restricted to biological process and performed using the topGO R package version 2.34.0 (Adrian A. and Jorg R.). Reported p-values were two-sided, and differences were considered significant when the p-value was less than 0.05. When applicable, multiple testing correction was performed using the false discovery rate method (FDR) (Benjamini and Hochberg, 1995), FDR below 0.05 was considered significant. All analyses were performed using R software version 3.5.2 (available at www.r-project.org) and Bioconductor version 3.8.

## Results

### Patients and survival

To identify carcinoma with mucinous differentiation, we used an ontology-based text mining approach on the available pathology reports (see “Methods”). A total of 902 patients (233 with mucinous features and 669 without mucinous features) representing six cancer types (95 colorectal adenocarcinomas; CRC, 36 invasive ductal breast carcinomas; BRCA, 36 lung adenocarcinomas; LUAD, 33 stomach adenocarcinomas; STAD, 18 cervical adenocarcinomas; CEAD and 15 pancreatic adenocarcinomas; PAAD) were included in this study (Supplementary Table 1). To reduce confounding factors, for each cancer type, each mucinous case was matched to three controls according to common cancer-type specific clinicopathological features (see “Methods”). Clinicopathological characteristics of carcinomas with mucinous differentiation (hereinafter referred to as “carcinomas-muc”) and non-mucinous carcinomas (hereinafter referred to as “controls”) are detailed in Supplement Table 1. We did not observe differences in overall survival (OS) or progression-free interval (PFI) within each cancer type, except for STAD-muc having a longer OS (median OS; 4.8 years vs 1.6 years, *P* = 0.03, adjusted hazard ratio, aHR = 0.4; 95% confidence interval, 95CI; 0.18–0.9, *P* = 0.015), and CEAD-muc having a median PFI of 3.1 vs. the median PFI was not reached in CEAD (*P* = 0.03) (aHR = 5.5, 95CI; 0.96–31.2, *P* = 0.04) (Supplementary Table 2 and Supplementary Fig. S1).

### Transcriptomic similarities across carcinomas with mucinous differentiation from different cancer types

**Figure 1:**
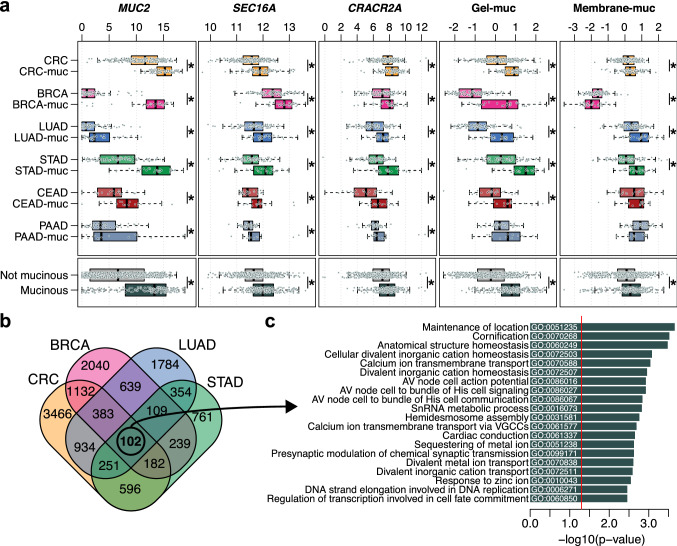
Transcriptomic similarities across carcinomas with mucinous differentiation from different cancer types. (**a**) Comparison of MUC2, SEC16A, CRACR2A, the gel-forming mucins signature (Gel-muc) and the membrane-bound mucins signature (Membrane-muc) between carcinomas-muc and controls for each cancer type (*; FDR < 0.05). Statistical significance was measured using the pairwise Mann–Whitney U tests adjusted for multiple comparisons. (**b**) Number of shared and unique differentially expressed genes between carcinoma-muc and control across each cancer type. (**c**) Top 20 enriched gene ontology terms associated with the 102 common differentially expressed genes. *CRC* colorectal adenocarcinoma, *CRC-muc* colorectal mucinous adenocarcinoma, *BRCA* infiltrating ductal carcinoma breast cancer, *BRCA-muc* mucinous carcinomas of the breast, *LUAD* lung adenocarcinoma, *LUAD-muc* mucinous (colloid) adenocarcinoma of the lung, *STAD* stomach adenocarcinoma, *STAD-muc* mucinous adenocarcinoma of the stomach, *CEAD* endocervical adenocarcinoma, *CEAD-muc* mucinous adenocarcinoma of endocervical type, *PAAD* pancreas-adenocarcinoma ductal type, *PAAD-muc* pancreas-colloid (mucinous non-cystic) carcinoma.

To better understand the intrinsic biology of carcinomas-muc, we performed a differential gene expression analysis between carcinomas-muc and their respective controls. For each cancer type, we identified a list of statistically significantly differentially expressed genes (7046, 4826, 4556, 2594, 2366, 478, respectively for CRC, BRCA, LUAD, STAD, CEAD and PAAD, Supplementary Table 3). The intersection of these lists revealed that three genes (*MUC2*, *CRACR2A*, *SEC16A*, Fig. 1a) were significantly up-regulated in carcinomas-muc of all tested cancer types compared to controls. We previously reported two different mucin gene expression signatures, composed of secreted gel-forming mucins and membrane-bound mucins, and observed that only the gel-forming signature was elevated in mucinous breast cancer^11^. Here, we observed that in every cancer type except PAAD, the secreted gel-forming mucins signature was statistically significantly upregulated in mucinous carcinoma compared to controls (Fig. 1a). Of note, the membrane-bound mucins signature was significantly up-regulated in CRC-muc, LUAD-muc and STAD-muc but downregulated in BRCA-muc. When restricting the analysis to the four largest cohorts (CRC, BRCA, LUAD and STAD), a total of 102 genes were consistently differentially expressed between carcinomas-muc and controls (Fig. 1b), a number that is statistically significantly higher than expected by chance (*P* < 0.001, based on Monte-Carlo simulations). *MUC5B,* another gel-forming mucin, was identified to be up-regulated in all four cancer types (Supplementary Fig. S2A). We previously demonstrated that expression of *MUC2* in mucinous breast cancer was regulated by DNA methylation^10,11^. We interrogated if a similar mechanism was present in other carcinomas-muc and found that the expression of *MUC2* was regulated by DNA methylation in CRC-muc, BRCA-muc and STAD-muc (Supplementary Fig. S2B,C and Supplementary Table 4). Gene ontology enrichment analysis using the 102 previously identified genes revealed enrichment of processes related to cellular divalent cation homeostasis and calcium (Ca++) transmembrane transport (Fig. 1c and Supplementary Fig. S2D). Taken together, these results support the notion that carcinomas with mucinous differentiation from different cancer types share transcriptomic similarities.

### The unique genomic landscape of carcinomas with mucinous differentiation across different cancer types

**Figure 2:**
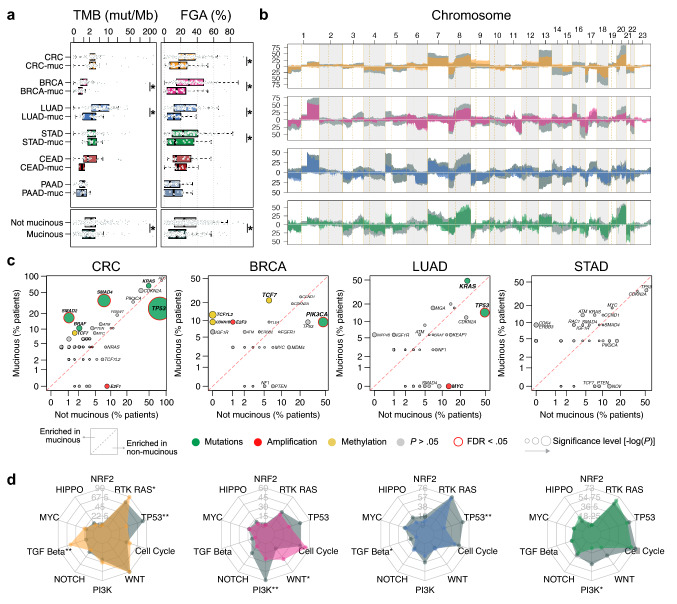
The unique genomic landscape of carcinomas with mucinous differentiation from different cancer types. (**a**) Comparison of TMB (left), FGA (middle) and the number of driver alterations (right) between carcinoma-muc and control for each cancer type (*; FDR < 0.05). Statistical significance was measured using the pairwise Mann–Whitney *U* tests adjusted for multiple comparisons. (**b**) Comparison of the CNAs frequencies between carcinoma-muc (colored) and control (grey) for each cancer type. (**c**) Comparison of the frequency of recurrent oncogenic alteration present in each cancer type. Statistical significance was measured using the Fisher’s exact test adjusted for multiple comparisons. (**d**) Percentages of patients with alterations in canonical oncogenic signaling pathways between carcinoma-muc (colored) and control (grey) for each cancer type (*P < 0.05, **FDR < 0.05). Note that the NRF2 pathway was not altered in PAAD. Statistical significance was measured using the Fisher’s exact test adjusted for multiple comparisons. *TMB* tumor mutation burden, *FGA* fraction of genome altered, *CRC* colorectal adenocarcinoma, *CRC-muc* colorectal mucinous adenocarcinoma, *BRCA* infiltrating ductal carcinoma breast cancer, *BRCA-muc* mucinous carcinomas of the breast, *LUAD* lung adenocarcinoma, *LUAD-muc* mucinous (colloid) adenocarcinoma of the lung, *STAD* stomach adenocarcinoma, *STAD-muc* mucinous adenocarcinoma of the stomach, *CEAD* endocervical adenocarcinoma, *CEAD-muc* mucinous adenocarcinoma of endocervical type, *PAAD* pancreas-adenocarcinoma ductal type, *PAAD-muc* pancreas-colloid (mucinous non-cystic) carcinoma.

To chart the somatic genomic landscape of carcinomas with mucinous differentiation, we systematically profiled broad genomic features, recurrent oncogenic alterations and pathway alterations in carcinomas-muc and controls. As previously shown^1^, we observed that CRC-muc is enriched for microsatellite unstable (MSI-H) tumors (26/73 (35.6%) vs. 27/215 (12.6%), *P* < 0.001). To reduce the heterogeneity of the cohort, we chose to exclude MSI-H cases (N = 75) in subsequent genomic analyses. When interrogating broad genomic features, we found that tumor mutational burden (TMB) was significantly lower in BRCA-muc and LUAD-muc and that fraction of genome altered (FGA), a measure for the degree of copy-number changes, was significantly lower in CRC-muc, BRCA-muc, LUAD-muc and STAD-muc than in controls (Fig. 2a). We also compared the number of driver alterations between carcinomas-muc and their non-mucinous counterparts within each cancer type. BRCA-muc and LUAD-muc had a significantly lower number of driver alterations, which is consistent with the lower TMB and FGA observations. Intriguingly, CRC-muc had a slightly higher number of driver alterations than CRC (Fig. 2a). Next, we compared the frequency of genome-wide copy number alterations (CNA) between carcinomas-muc and controls in CRC, BRCA, LUAD and STAD. Compared to their control counterparts, carcinomas-muc had a lower frequency of CNA, evenly distributed across the genome (Fig. 2b). Then, for each cohort, we identified recurrent oncogenic alterations and compared their frequencies in controls and carcinomas-muc. Compared to their control counterparts, we observed a lower frequency of *TP53* mutations in CRC-muc and LUAD-muc (12/48 (25%) vs. 146/189 (77.2%), FDR < 0.001 and 5/35 (14.3%) vs. 49/101 (48.5%), FDR = 0.02, respectively), a higher frequency of *SMAD4* and *SMAD2* mutations in CRC-muc (17/48 (35.4%) vs. 14/189 (7.4%), FDR = 0.0001 and 8/48 (16.7%) vs. 2/189 (1.1%), FDR = 0.001, respectively) and a lower frequency of *PIK3CA* mutations in BRCA-muc (3/32 (9.4%) vs. 45/98 (45.9%), FDR = 0.006). Of note, we detected a lower frequency of *TP53* mutation in BRCA-muc and PAAD-muc, a higher frequency of *KRAS* mutation in CRC-muc and LUAD-muc and a higher frequency of methylation silencing of *TCF7* in CRC-muc and BRCA-muc and *TCF7L2* and *CDKN1B* in BRCA-muc, although these observations did not reach statistical significance after correcting for multiple testing (Fig. 2c). Next, we compared the frequency of ten canonical oncogenic pathways commonly altered in cancer (RTK-RAS, p53, cell cycle, β-catenin/Wnt, PI-3-Kinase/Akt, Notch, TGFβ signaling, Myc, Hippo and Nrf2, as described in^15^) in controls and carcinomas-muc. In CRC-muc, we observed a lower frequency of p53 pathway alteration and a higher frequency of TGFβ signaling and RTK-RAS pathways alterations (19/48 (39.6%) vs. 153/189 (81%) FDR < 0.001, 28/48 (58.3%) vs. 35/189 (18.5%) FDR < 0.001, 43/48 (89.6%) vs. 140/189, FDR = 0.07). In BRCA-muc, we observed a lower frequency of PI-3-Kinase/Akt pathway alterations and a higher frequency of β-catenin/Wnt pathway alterations (8/32 (25%) vs. 58/98 (59.1%), *P* = 0.001, FDR = 0.01 and 11/32 (34.4%) vs. 13/98 (13.3%), *P* = 0.02, FDR = 0.08). In LUAD-muc, we observed a lower frequency of p53 and TGFβ signaling pathways alterations (8/35 (22.9%) vs. 63/101 (62.4%), *P* < 0.001, FDR < 0.001 and 0/35 (0%) vs. 12/101 (11.8%), *P* = 0.03, FDR = 0.18). Finally, in STAD-muc, we observed a lower frequency of PI-3-Kinase/Akt pathway alterations (2/22 (9%) vs. 28/69 (40.6%), *P* = 0.008, FDR = 0.07) (Fig. 2d). These data indicate that the genomic landscape of carcinomas-muc is different from that of their control counterparts. Carcinomas with mucinous differentiation, however, share genomic similarities across different cancer types, such as quieter copy-number alteration profiles in CRC-muc, BRCA-muc, LUAD-muc and STAD-muc, a lower frequency of p53 pathway alterations in CRC-muc and LUAD-muc and a lower frequency of PIK3CA pathway alterations in BRCA-muc and STAD-muc.

## Discussion

To our knowledge, this is the first systematic attempt to characterize carcinomas with mucinous features across different cancer types. In this study, we have demonstrated that the up-regulation of *MUC2*, *SEC16A* and *CRACR2A* is the common denominator of all carcinomas with mucinous differentiation studied here. The up-regulation of *MUC2*, the most abundant secreted mucin, has been previously shown in different cancer types^1,8,9^. Several studies have reported a possible role of DNA methylation in regulating the expression of some gel-forming mucins^22^ and the epigenetic regulation of *MUC2* has been shown in mucinous colorectal cancer and mucinous breast cancer^10,11,23^. We demonstrated that *MUC2* expression is also regulated by DNA methylation in stomach cancer. The fact that we did not find an association between *MUC2* expression and DNA methylation in LUAD, CEAD and PAAD might be due to low statistical power or other mechanisms of mRNA expression regulation such as histone methylation^24^. Nevertheless, our results suggest that up-regulation of *MUC2* and the gel-forming mucins signature might be considered as pan-cancer biomarkers of mucinous histology. *SEC16A* encodes a protein required for protein trafficking to the cell membrane^25^. We hypothesize that carcinomas-muc up-regulates *SEC16A* to sustain mucin secretion. Differential gene expression analysis revealed up-regulation of genes related to divalent cation homeostasis and Ca++ transmembrane transport such as *CRACR2A,* encoding a rab GTPase Ca++ binding protein and acting as a cytoplasmic calcium-sensor*.* Interestingly, it has been shown that packing and releasing of gel-forming *MUC2* is a pH-dependent mechanism that relies on Ca++ release regulation^26^. We show that, independent of cancer type, carcinomas with mucinous differentiation not only display elevated expression of *MUC2* but also complementary genes involved in *MUC2* packing, folding and transport.

At the genomic level, we found that a flat CNA landscape was a common trait across CRC-muc, BRCA-muc, LUAD-muc and STAD-muc. Thus, we hypothesize that carcinomas-muc are less likely driven by copy number alterations. Higher frequencies of *SMAD4, BRAF* and *KRAS* mutations have been previously reported in CRC-muc^1,2,27^. Here, we confirmed these observations and found that CRC-muc was also associated with a higher frequency of *SMAD2* and lower frequency of *TP53* mutations. The slightly higher median number of driver alterations observed in CRC-muc might be explained by the higher frequency of mutation in genes belonging to TGF-Beta and RTK-RAS pathways in CRC-muc. We and others have previously reported a lower TMB, FGA and lower frequency of *PIK3CA* mutation in mucinous breast cancer^10,11^. Here we found that BRCA-muc were associated with a higher frequency of β-catenin/Wnt pathway alterations, mostly caused by epigenetic silencing of *TCF7L2* and *TCF7*. We have also observed a higher frequency of *KRAS* mutations and lower frequency of *TP53* mutations in LUAD-muc, consistent with previous findings^3,28^.

Our study has several limitations. First, the definition of mucinous carcinomas varies by cancer types and includes pure as well as mixed mucinous carcinomas, but due to the pan-cancer nature of this study and for practical reasons, we choose to include any carcinoma with mucinous differentiation. Of note, not all HE slides were available to be centrally reviewed. It should also be noted that the sample size was relatively low for CEAD and PAAD hence negative conclusions related to these cohorts should be interpreted with caution, given the limited statistical power. Finally, only samples that were part of the TCGA were analyzed and future studies that include ovarian and appendiceal cancer are needed to investigate if their biology are similar to other mucinous carcinomas.

In conclusion, here we provide important novel insights into the biology of carcinomas with mucinous differentiation suggesting transcriptional and genomic similarities across different cancer types and alternative oncogenic pathway alterations underlying their pathogenesis, paving the way for a more biologically relevant taxonomy for these cancers.

## Supplementary Information

Supplementary Information 1.

Supplementary Table 1.

Supplementary Table 2.

Supplementary Table 3.

Supplementary Table 4.

Supplementary Table 5.
