# Supplementary material for: The genomic landscape of carcinomas with mucinous differentiation
Source: Sci Rep. 2021 May 4;11:9478. doi: 10.1038/s41598-021-89099-2 (PMC8097060; doi:10.1038/s41598-021-89099-2)
Supplement: Supplementary file 1 — Supplementary Information 1. [file 41598_2021_89099_MOESM1_ESM.pdf]

SUPPLEMETARY MATERIAL

The genomic landscape of carcinomas with mucinous differentiation

Bastien Nguyen, Francisco Sanchez-Vega, Christopher J. Fong, Walid K. Chatila, Amir Momeni Boroujeni, Fresia Pareja, Britta Weigelt, Christos Sotiriou, Denis Larsimont, Jorge S. Reis-Filho, Christine Desmedt, Nikolaus Schultz

SUPPLEMETARY TABLE

Supplementary Table 1. List of samples included in this study with detailed clinicopathological features.

Supplementary Table 2. Clinicopathological features of cancer with mucinous differentiation (-muc) and their non-mucinous counterparts (control) for each cancer type.

Supplementary Table 3. Output from the differential expression analyses between mucinous and their control counterpart for each cancer type from DESeq2.

Supplementary Table 4. Output from the differential methylation analyses between mucinous and their control counterpart for each cancer type from dmpFinder, data are available at: https://doi.org/10.6084/m9.figshare.12209951.v1.

Supplementary Table 5. Comparison of the frequency of recurrent oncogenic alteration between mucinous and their control counterpart for each cancer type.

Supplementary Table 2. Clinicopathological features of cancer with mucinous differentiation (-muc) and their non-mucinous counterparts (control) for each cancer type.

Supplementary Table 2. Clinicopathological features of cancer with mucinous differentiation (-muc) and their non-mucinous counterparts (control) for each cancer type

		CRC	CRC-muc	*P*	BRCA	BRCA-muc	*P*	LUAD	LUAD-muc	*P*	STAD	STAD-muc	*P*	CEAD	CEAD-muc	*P*	PAAD	PAAD-muc	*P*
**N**		285	95		108	36		108	36		99	33		24	18		45	15	
**Age at diagnosis***	**<= median**	140 (49.1%)	50 (52.6%)		38 (35.2%)	14 (38.9%)		47 (43.5%)	16 (44.4%)		53 (53.5%)	18 (54.5%)		13 (54.2%)	8 (44.4%)		22 (48.9%)	9 (60%)	
	**> median**	145 (50.9%)	45 (47.4%)	0.64	70 (64.8%)	22 (61.1%)	0.84	61 (56.5%)	20 (55.6%)	1	46 (46.5%)	15 (45.5%)	1	11 (45.8%)	10 (55.6%)	0.755	23 (51.1%)	6 (40%)	0.65
**Year of diagnosis****	**<= median**	166 (58.2%)	57 (60%)		43 (39.8%)	14 (38.9%)		57 (52.8%)	19 (52.8%)		60 (60.6%)	18 (54.5%)		15 (62.5%)	9 (50%)		33 (73.3%)	10 (66.7%)	
	**> median**	119 (41.8%)	38 (40%)	0.86	65 (60.2%)	22 (61.1%)	1	51 (47.2%)	17 (47.2%)	1	39 (39.4%)	15 (45.5%)	0.68	9 (37.5%)	9 (50%)	0.621	12 (26.7%)	5 (33.3%)	0.74
**Gender**	**Female**	145 (50.9%)	48 (50.5%)		108 (100%)	36 (100%)		43 (39.8%)	15 (41.7%)		17 (17.2%)	5 (15.2%)		24 (100%)	18 (100%)		7 (15.6%)	3 (20%)	
	**Male**	140 (49.1%)	47 (49.5%)	1	0 (0%)	0 (0%)	/	65 (60.2%)	21 (58.3%)	1	82 (82.8%)	28 (84.8%)	1	0 (0%)	0 (0%)	/	38 (84.4%)	12 (80%)	0.7
**pT**	**T1**	4 (1.4%)	2 (2.1%)		23 (21.3%)	8 (22.2%)		22 (20.4%)	7 (19.4%)		2 (2%)	1 (3%)		15 (62.5%)	10 (55.6%)		3 (6.7%)	2 (13.3%)	
	**T2**	32 (11.2%)	12 (12.6%)		71 (65.7%)	21 (58.3%)		62 (57.4%)	19 (52.8%)		12 (12.1%)	5 (15.2%)		7 (29.2%)	5 (27.8%)		10 (22.2%)	3 (20%)	
	**T3**	211 (74%)	67 (70.5%)		5 (4.6%)	4 (11.1%)		21 (19.4%)	9 (25%)		59 (59.6%)	18 (54.5%)		1 (4.2%)	1 (5.6%)		32 (71.1%)	9 (60%)	
	**T4**	38 (13.3%)	14 (14.7%)	0.83	9 (8.3%)	3 (8.3%)	0.54	3 (2.8%)	1 (2.8%)	0.89	26 (26.3%)	9 (27.3%)	0.87	0 (0%)	1 (5.6%)	0.926	0 (0%)	0 (0%)	0.31
**pN**	**N0**	179 (62.8%)	58 (61.1%)		54 (50%)	18 (50%)		73 (67.6%)	23 (63.9%)		15 (15.2%)	6 (18.2%)		17 (70.8%)	8 (44.4%)		11 (24.4%)	5 (33.3%)	
	**N1**	55 (19.3%)	19 (20%)		48 (44.4%)	16 (44.4%)		21 (19.4%)	8 (22.2%)		40 (40.4%)	12 (36.4%)		5 (20.8%)	3 (16.7%)		33 (73.3%)	9 (60%)	
	**N2**	51 (17.9%)	18 (18.9%)	0.95	4 (3.7%)	1 (2.8%)		14 (13%)	5 (13.9%)	0.88	42 (42.4%)	14 (42.4%)		/	/		/	/	
	**NX**	/	/		2 (1.9%)	1 (2.8%)	1	/	/		2 (2%)	1 (3%)	0.9	2 (8.3%)	7 (38.9%)	0.052	1 (2.2%)	1 (6.7%)	0.43
**pM**	**M0**	220 (77.2%)	73 (76.8%)		80 (74.1%)	27 (75%)		78 (72.2%)	26 (72.2%)		94 (94.9%)	31 (93.9%)		23 (95.8%)	16 (88.9%)		24 (53.3%)	8 (53.3%)	
	**M1**	35 (12.3%)	10 (10.5%)		/	/		2 (1.9%)	1 (2.8%)		5 (5.1%)	2 (6.1%)	1	1 (4.2%)	2 (11.1%)	0.57	/	/	
	**MX**	30 (10.5%)	12 (12.6%)	0.79	28 (25.9%)	9 (25%)	1	28 (25.9%)	9 (25%)	1	/	/		/	/	/	21 (46.7%)	7 (46.7%)	1
**Site**	**Colon**	209 (73.3%)	76 (80%)		/	/		/	/		/	/		/	/		/	/	
	**Rectum**	76 (26.7%)	19 (20%)	0.24	/	/		/	/		/	/		/	/		/	/	
**Laterality**	**Left**	111 (38.9%)	31 (32.6%)		/	/		/	/		/	/		/	/		/	/	
	**Right**	107 (37.5%)	45 (47.4%)		/	/		/	/		/	/		/	/		/	/	
	**NA**	67 (23.5%)	19 (20%)	0.24	/	/		/	/		/	/		/	/		/	/	
**ER status**	**Negative**	/	/		3 (2.8%)	1 (2.8%)		/	/		/	/		/	/		/	/	
	**Positive**	/	/		101 (93.5%)	34 (94.4%)		/	/		/	/		/	/		/	/	
	**[Not Evaluated]**	/	/		4 (3.7%)	1 (2.8%)	1	/	/		/	/		/	/		/	/	
**PR status**	**Negative**	/	/		11 (10.2%)	4 (11.1%)		/	/		/	/		/	/		/	/	
	**Positive**	/	/		93 (86.1%)	31 (86.1%)		/	/		/	/		/	/		/	/	
	**[Not Evaluated]**	/	/		4 (3.7%)	1 (2.8%)	1	/	/		/	/		/	/		/	/	
**HER2 status**	**Equivocal**	/	/		18 (16.7%)	7 (19.4%)		/	/		/	/		/	/		/	/	
	**Negative**	/	/		73 (67.6%)	24 (66.7%)		/	/		/	/		/	/		/	/	
	**Positive**	/	/		6 (5.6%)	2 (5.6%)		/	/		/	/		/	/		/	/	
	**[Not Evaluated]**	/	/		11 (10.2%)	3 (8.3%)	0.97	/	/		/	/		/	/		/	/	
**Tobacco smoking history**	**Current reformed smoker**	/	/		/			70 (64.8%)	24 (66.7%)		/	/		2 (9.1%)	2 (11.8%)		/	/	
	**Current smoker**	/	/		/			14 (13%)	4 (11.1%)		/	/		4 (18.2%)	3 (17.6%)		/	/	
	**Lifelong Non-smoker**	/	/		/			22 (20.4%)	7 (19.4%)		/	/		16 (72.7%)	12 (70.6%)		/	/	
	**[Unknown]**	/	/		/			2 (1.9%)	1 (2.8%)	1	/	/		/	/	1	/	/	
**HPV status**	**Negative**	/	/		/			/	/		/	/		4 (16.6%)	4 (22.2%)		/	/	
	**Positive**	/	/		/			/	/		/	/		18 (75.0%)	14 (77.8%)		/	/	
	**Indeterminate**	/	/		/			/	/		/	/		2 (8.3%)	0 (0.0%)	1	/	/	

***P*; P-value derived form the χ2 test or the Fisher's exact test when appropriate**

***Median age at diagnosis (years); 67, 58, 66, 68, 44.5 and 65 respectively for CRAD, BRCA, LUAD, STAD, CEAD, PAAD**

****Median year of diagnosis; 2009, 2009, 2010, 2011, 2011, 2012 respectively for CRAD, BRCA, LUAD, STAD, CEAD, PAAD**

CRC, colorectal adenocarcinoma; CRC-muc, colorectal mucinous adenocarcinoma; BRCA, infiltrating ductal carcinoma breast cancer; BRCA-muc, mucinous carcinomas of the breast; LUAD, lung adenocarcinoma; LUAD-muc, mucinous (colloid) adenocarcinoma of the lung; STAD, stomach adenocarcinoma; STAD-muc, mucinous adenocarcinoma of the stomach; CEAD, endocervical adenocarcinoma; CEAD-muc, mucinous adenocarcinoma of endocervical type; PAAD, pancreas-adenocarcinoma ductal type; PAAD-muc, pancreas-colloid (mucinous non-cystic) carcinoma.

SUPPLEMENTARY FIGURES

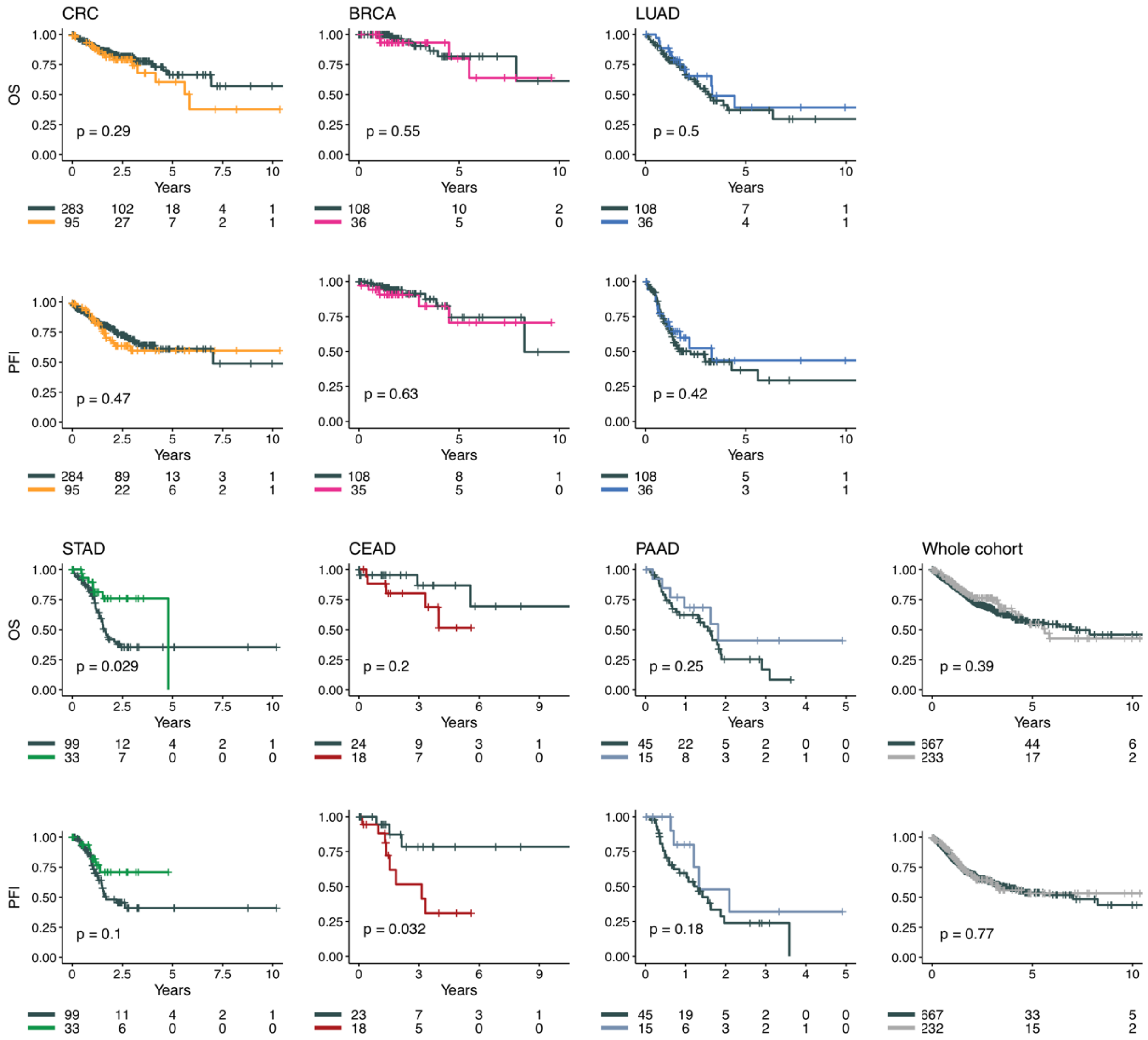

Supplementary Figure S1. Comparison of overall survival (OS) and progression-free interval (PFI) between carcinoma-muc (colored) and control (grey) for each cancer type and the whole cohort. p; p-value derived from the log-rank test.

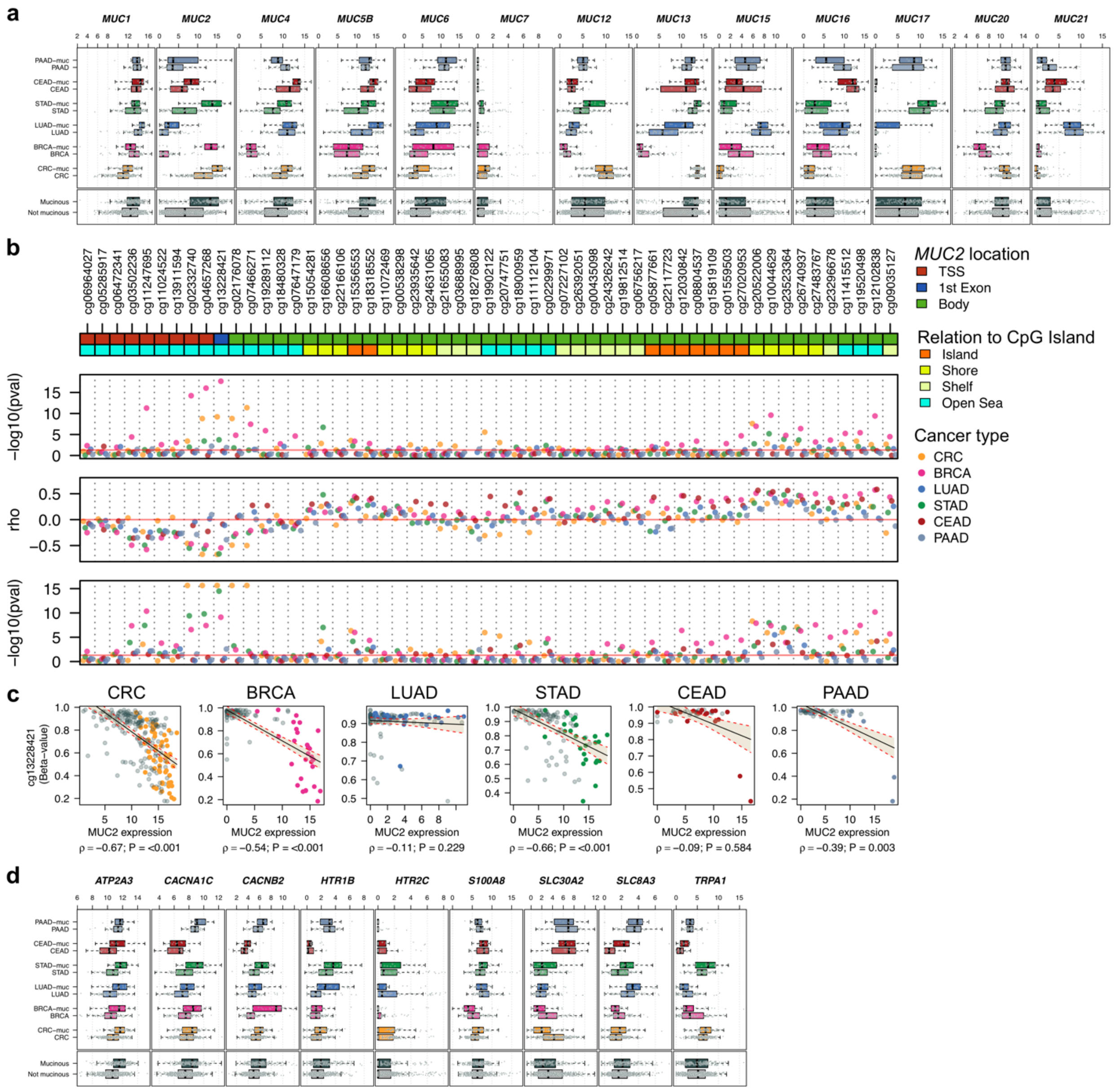

Supplementary Figure S2. a, Comparison of the normalized expression of genes (log2(FPKM+1)) belonging to the mucin gene family between carcinoma-muc and control for each cancer type. Only *MUC2* and *MUC5B* are commonly differentially expressed between carcinomas-muc and controls in the four largest cohorts (DESeq2 FDR < 0.05 in CRC, BRCA, LUAD and STAD). **b**, Epigenetic regulation of *MUC2* in carcinomas-muc. Each column represents the 55 CpGs mapped to *MUC2*. The upper panel shows the significance level of the

differentially methylated positions between carcinoma-muc and control for each cancer type (p-value derived for dmpFinder in the minfi package). The middle panel shows the Spearman's rho correlation coefficient between the CpG and *MUC2* expression for each cancer type. The lower panel shows the significance level the Spearman's correlation between the CpG beta-value and MUC2 expression for each cancer type. **c**, Spearman's correlation between cg13228421 beta value and *MUC2* expression for each cancer type. Each dot is a sample, colored dots represent carcinomas-muc, grey dots are the controls. **d**, Comparison of the normalized expression of genes (log2(FPKM+1)) annotated as cellular divalent inorganic cation homeostasis (GO:0072503) and significantly differentially expressed between carcinoma-muc and control for each cancer type (DESeq2 FDR < 0.05 in CRC, BRCA, LUAD and STAD). CRC, colorectal adenocarcinoma; CRC-muc, colorectal mucinous adenocarcinoma; BRCA, infiltrating ductal carcinoma breast cancer; BRCA-muc, mucinous carcinomas of the breast; LUAD, lung adenocarcinoma; LUAD-muc, mucinous (colloid) adenocarcinoma of the lung; STAD, stomach adenocarcinoma; STAD-muc, mucinous adenocarcinoma of the stomach; CEAD, endocervical adenocarcinoma; CEAD-muc, mucinous adenocarcinoma of endocervical type; PAAD, pancreas-adenocarcinoma ductal type; PAAD-muc, pancreas-colloid (mucinous non-cystic) carcinoma.
